# Long-term in situ permafrost thaw effects on bacterial communities and potential aerobic respiration

**DOI:** 10.1038/s41396-018-0176-z

**Published:** 2018-06-06

**Authors:** Sylvain Monteux, James T. Weedon, Gesche Blume-Werry, Konstantin Gavazov, Vincent E. J. Jassey, Margareta Johansson, Frida Keuper, Carolina Olid, Ellen Dorrepaal

**Affiliations:** 1 Climate Impacts Research Centre (CIRC), Department of Ecology and Environmental Sciences, Umeå Universitet, 981 07 Abisko, Sweden; 2 Systems Ecology, Department of Ecological Sciences, Vrije Universiteit Amsterdam, 1081 HV Amsterdam, The Netherlands; 3 PLECO, Department of Biology, University of Antwerp, 2610 Wilrijk, Belgium; 4 Federal Institute for Forest, Snow and Landscape Research WSL, CH-1015 Lausanne, Switzerland; 5 Functional Ecology and Environment Laboratory (ECOLAB), Department of Biology and Geosciences, UMR 6245 Université Toulouse III Paul Sabatier, 31062 Toulouse cedex 09, France; 6 Department of Physical Geography and Ecosystem Science, Lund Universitet, 223 62 Lund, Sweden; 7 INRA, AgroImpact UR1158, Site Laon, 02000 Barenton Bugny, France

## Abstract

The decomposition of large stocks of soil organic carbon in thawing permafrost might depend on more than climate change-induced temperature increases: indirect effects of thawing via altered bacterial community structure (BCS) or rooting patterns are largely unexplored. We used a 10-year in situ permafrost thaw experiment and aerobic incubations to investigate alterations in BCS and potential respiration at different depths, and the extent to which they are related with each other and with root density. Active layer and permafrost BCS strongly differed, and the BCS in formerly frozen soils (below the natural thawfront) converged under induced deep thaw to strongly resemble the active layer BCS, possibly as a result of colonization by overlying microorganisms. Overall, respiration rates decreased with depth and soils showed lower potential respiration when subjected to deeper thaw, which we attributed to gradual labile carbon pool depletion. Despite deeper rooting under induced deep thaw, root density measurements did not improve soil chemistry-based models of potential respiration. However, BCS explained an additional unique portion of variation in respiration, particularly when accounting for differences in organic matter content. Our results suggest that by measuring bacterial community composition, we can improve both our understanding and the modeling of the permafrost carbon feedback.

## Introduction

Northern hemisphere permafrost soils and their overlying active (seasonally thawing) layer store 1035–1580 Pg of organic carbon, about 28% of which is found in peat deposits [1, 2]. This carbon stock is protected from decomposition due to limited microbial activity in frozen soils [3, 4]. Climate change-induced thawing of permafrost soils stimulates decomposition of this carbon, potentially causing a positive feedback to warming [2, 5]. However, it remains uncertain how permafrost thaw will affect soil organic matter (SOM) decomposition in the long term, and how this relates to changes in other potential drivers of SOM decomposition, such as bacterial communities and plant root distribution.

While higher temperatures increase soil respiration in laboratory incubations [6], field studies have shown contrasting results, with increased but also reduced responses of soil respiration to experimental warming and permafrost thaw in the longer term (e.g. [7, 8]). Long-term decreases in soil respiration have variously been attributed to depletion of labile carbon substrates or microbial acclimation [9]. Decomposition in natural thawing permafrost soil may therefore be controlled not only by increases in temperature but also by various associated changes in the ecosystem. Permafrost thaw can, for example, alter other soil abiotic conditions, such as nutrient availability [10], which can in turn affect decomposition rates [11]. In the longer term, active layer-deepening can favor deeper-rooting plant species [12, 13], which might increase SOM decomposition through rhizodeposition of fresh carbon (priming effects [14–16]). Deep roots in permafrost peatlands might stimulate SOM decomposition through increased oxygen availability if they harbor aerenchymae [17], as anoxia partly inhibits SOM decomposition in peatlands [18]. Moreover, changes in abiotic soil properties or rooting patterns may affect decomposition indirectly through altering microbial community structure [19, 20]. In the long term, SOM decomposition is therefore likely altered by permafrost thaw due to changes in temperature, root density, abiotic soil conditions, or microbial community structure, but, so far, the extent and underlying ecological interactions remain unclear.

Microorganisms are important decomposers, especially in deeper soil layers, which larger soil fauna cannot access [21, 22]. Nonetheless, the importance of microbial community structure for decomposition rates is controversial [23]. On the one hand, soil microbial communities are often considered to exhibit high functional redundancy with regard to decomposition, making their composition unimportant for determining process rates [24]. On the other hand, microbial community structure may affect decomposition in some systems, e.g., when microbial diversity is very low [25]. Deeper, permanently frozen soil layers exhibit lower microbial biomass, diversity, and functional potential than the active layer, due to long-term adaptation to frozen conditions [26, 27]. Bacteria have higher abundance than archaea and fungi in permafrost subsoil systems [26, 28, 29] and are important contributors to respiration of peatland soils, especially at high water content [30, 31]. Bacterial community structure (BCS; sensu taxonomic composition) could therefore be an important determinant of decomposition in thawing permafrost soils, but so far, this link has not been directly evaluated.

BCS may be affected by warming-induced changes to plant community composition and rooting behavior [12, 32, 33], through altered rhizosphere extent and properties [20, 34]. These effects will be strongest in the newly thawed permafrost at the base of the active layer, where no roots were previously present. Thawing permafrost soils will also experience physico-chemical changes: phase transition in the newly thawed permafrost will remove the long-term freezing constraints (e.g., low water availability and energy input) with potentially large effects on bacterial communities [28, 35]. Climate change and permafrost thaw are thus likely to modify bacterial communities through alterations in soil abiotic factors and plant root distribution, with the strongest effects in the newly thawed soil layers.

Common approaches to study the microbial ecology of thawing permafrost are natural thaw gradients (e.g. [26]) and laboratory incubations (e.g. [28]). Natural thaw gradients provide realistic proxies for future effects of climate change, but the history of deep-thaw sites may not resemble current shallow-thaw sites. BCS can vary strongly with depth within a soil profile [36] and the thawing of permafrost induces varying soil subsidence and compaction depending on ice content [37]. Therefore, ensuring that the communities compared along a presumed thaw gradient were not different before permafrost thaw occurred can be challenging. Such potential artifacts are avoided by repeated sampling from thawing and incubating permafrost samples but in vitro approaches ignore indirect effects of thawing, such as through soil fauna and root colonization. In contrast, inducing permafrost thaw in the field, e.g., by experimentally increasing winter insulation with snow fences to simulate winter climate change [38, 39], allows for the detection of these longer-term indirect effects of thawing. Such an experimental setup also allows for more precision in sampling soil from a given depth as subsidence can be monitored. Furthermore, by careful selection of plots and randomization of treatments initially identical microbial communities are ensured, precluding the potential confounding effects inherent to spatial gradients.

We investigated how the combined direct and indirect effects of long-term in situ permafrost thaw through experimental winter-warming modifies the BCS and potential soil respiration across the active layer and upper permafrost soil, and studied whether and how these responses were linked. We hypothesized that:

(a) Decadal in situ winter-warming and associated permafrost thaw will modify BCS across active layer and permanently frozen soil, most strongly at the depth of the newly thawed and recently root-colonized soil.

(b) Potential respiration will decrease with decadal winter-warming in the active layer and increase in the newly thawed permafrost soil. This is because of long-term depletion of labile carbon substrates after a decade of higher temperatures in the active layer. In contrast, the intermediate layer has only thawed for a short duration at the end of each growing season, so we expect increased respiration as a result of changes in BCS (e.g., by shifts toward communities with higher functional diversity).

(c) Including BCS—i.e., phylogeny-informed taxonomic composition—and plant root density will improve soil chemistry-based predictions of potential respiration across active layer and thawing permafrost soil.

## Materials and methods

### Deep-thaw experiment

The study site is located in the Storflaket palsa peatland close to Abisko, Northern Sweden (68.346N, 18.971E), of which active layer thickness (ALT) was 69.3 ± 3.7 cm in 2015, and water table depth was 30.2 ± 2.8 cm in October 2013 (mean ± SE). The peatland is dominated by peat moss (*Sphagnum* spp.), *Eriophorum vaginatum* L., *Vaccinium vitis-idaea* L., *Andromeda polifolia* L., *Betula nana* L., *Empetrum nigrum* L. and *Rubus chamaemorus* L. Since 2005, 10 m long and 1 m high snow fences were erected each winter in six randomly chosen plots out of 12 (10 × 10 m), increasing mean winter snow accumulation 2.6-fold (16–24 cm), winter soil temperature (at 15 cm) by 1.5 °C, and ALT by 29 cm (in 2015, data not shown), and are further referred to as “deep thaw”. The remaining six plots, with ambient snow accumulation (6–9 cm) served as controls. Effects include an increase in *E. vaginatum* growth and cover and in soil subsidence (average 24 cm in 2012 compared to 2005, vs 5 cm in control plots) causing seasonal ponds in some deep-thaw plots. Methane emissions were nonsignificant within or outside of the deep-thaw plots (0.09 ± 0.08 and 0.1 ± 0.18 mg CH_4_/m/h, respectively [40]), therefore we did not investigate methane dynamics. More details are found in ref. [41].

### Sampling design and sampling for BCS, plants, and abiotic analyses

We compared subsoil carbon and microbial dynamics around the thawfront at three depths: active layer (above the thawfront but below the water table in both deep-thaw and control plots, ca. 55 cm); intermediate layer (frozen in control plots, seasonally thawed in deep-thaw plots, ca. 70 cm); and permafrost layer (perennially frozen in both deep-thaw and control plots, at least 10 cm below the thawfront, 100–125 cm).

Two soil cores per plot were collected in September 2015, around the time of maximum ALT [41], using a peat-corer for thawed soil (11 × 11.4 cm, Eijkelkamp, The Netherlands), and a custom-made gas-powered fluid-less concrete drill for permafrost soil (10.2 cm diameter). As the ALT varies with distance to the fences within deep-thaw plots, we selected spots with ALT > 80 cm for coring, but we did not use ALT criteria for the cores in control plots (mean ± SE for control and deep-thaw cores were 60.1 ± 2.4 and 90.2 ± 6.4 cm, respectively; *P* < 0.001, *n* = 12). Frozen soil cores were rinsed with sterile deionized water to limit drilling contamination between soil layers, wrapped in plastic foil and stored in a cooler box for up to 3 h.

One set of cores was thawed at 2 °C after which 2 g soil was sampled for DNA extraction with ethanol-cleaned forceps, at least 3 cm from the core surface to minimize contamination, from the three aforementioned depths.

Three 30-cm segments were taken encompassing these sampling depths (i.e., 30–60, 60–90, >90 cm), and about 1 L (estimated by water displacement) of each core segment was used for manually collecting coarse roots (>0.5 mm), which were dried at 60 °C and weighed to calculate density of roots in the soil (g dry weight roots/L soil). Due to sampling errors, one replicate of the intermediate layer in deep-thaw plots was absent from this set of cores, and therefore excluded from analyses including BCS and root density.

The second set of cores was stored at −20 °C until April 2016, then thawed at 4 °C and subsamples were taken for soil chemistry analyses and aerobic incubations. These subsamples were homogenized by sieving through an ethanol-cleaned 2 mm sieve. Homogenized soil aliquots were used to measure potential aerobic respiration (see below), as an indicator of the vulnerability of soil carbon. Further, we used ~9 g subsamples to determine gravimetric water content (60 °C, 24 h) and organic matter content (OMC, 475 °C, 2 h [42]). Carbon content (g C/g dry soil) was analyzed using a NCS 2500 elemental analyzer (CE Instruments, Milan, Italy). Another 3 g (fresh weight) were shaken in 40 mL of sterile deionized water for 2 h and filtered. pH of the filtrate was measured on a MP220 pH meter (Mettler-Toledo, Greifensee, Switzerland) and NH_4_^+^ and (NO_3_^−^ + NO_2_^−^) were quantified on a FIAstar 5000 Autoanalyzer (FOSS Analytics, Hilleroed, Denmark). Nitrate and nitrite concentrations were below detection limit (ca. 12.2 µg NO_3_^−^-N/g dry soil) in most samples and are therefore not discussed further.

### Soil incubations

For each of the six replicates at three sampling depths in the two treatments, two aliquots of 20 g (fresh weight) homogenized soil were put into 250 mL glass jars closed with rubber septa and incubated in dark culture chambers at either 11 or 21 °C. Headspace air (10 mL) was sampled with an airtight glass syringe and CO_2_ concentrations were measured with an infrared gas analyzer (EGM-4, PP Systems, Amesbury, Massachusetts), using the instrument’s internal calibration and static sampling mode, at 1–5 days’ intervals for 1 month. When one sample reached ≥10 000 ppm, all jars were flushed with 400 ppm CO_2_ synthetic air, long enough to lower the headspace CO_2_ concentration to 400 ppm. One month after homogenization, respiration rates stabilized and four more measurements were taken, each time starting at 400 ppm (achieved by flushing as described above) and continuing for 1–2 days. These four measurements were used to calculate CO_2_ production rates (*τ*) as follows:

$$\tau _{\left( {1 - 2} \right)} = \dfrac{{n_2 - n_1}}{{\left( {\Delta _t} \right)_{1 - 2}}}$$ with $$n_i = \left[ {{\mathrm{CO}}_2} \right]_i \times \left( {P_iV/RT} \right)$$

where (Δ_*t*_)_1–2_ is the time interval between flushing (_1_) and measurement (_2_), _*Pi*_ is atmospheric pressure at flushing or measurement time (data from Abisko Naturvetenskapliga Station), *V* the headspace volume, *R* the ideal gas constant, and *T* the incubation temperature. The four rates were then averaged to estimate the potential aerobic respiration rates (hereafter respiration).

Temperature sensitivity of respiration (*Q*_10_ [43]) was calculated for each replicate soil sample based on these averaged values at 11 and 21 °C. Potential respiration rates were expressed either per soil dry weight (bulk respiration) or per organic matter dry weight (intrinsic respiration).

### Bacterial communities

DNA was extracted using a PowerSoil DNA Extraction Kit (Qiagen, Venlo, The Netherlands), following the manufacturer’s instructions. DNA was quantified using QuantIT dsDNA assay (Thermo Scientific, Waltham, Massachusetts, SI Fig S1). The DNA extracts were diluted to 5 ng/µL with nuclease-free water. Samples with concentrations <10 ng/µL were diluted 1:1.

The V3 region of the 16S ribosomal RNA gene was targeted in PCR amplification (341F; 518R, SI Table S1) to characterize BCS [44], and quality of amplification was visually checked by gel electrophoresis. Amplicons were cleaned and normalized using SequalPrep Normalization Plate Kit (Thermo Scientific), then pooled, and further purified using a QIAquick Gel Extraction Kit (Qiagen, Venlo, The Netherlands). The resulting pooled library was sequenced on an Illumina MiSeq with V2 chemistry and 2 × 150 bp paired-end reads (BioSample accession numbers SAMN07445364–SAMN07445398 and SAMN07445422).

All bioinformatics scripts are available as a Jupyter notebook at https://bitbucket.org/smonteux/monteux_deep_thaw. Merging, quality filtering, and 97% de novo operational taxonomic unit (OTU) clustering were performed with VSEARCH v1.10.2 [45], chimera removal with UCHIME, and the GOLD database [46, 47]. OTUs that were abundant in the technical control sample (>5%) were removed, alike in ref. [48]. PyNAST, FastTree, the RDP-naive Bayesian classifier, and Greengenes 13.8 database were used in QIIME v1.9.1 [49–52] to obtain OTUs taxonomy, filtering out OTUs present in <10% of the samples to exclude highly variable OTUs that may inflate the number of differentially abundant OTUs (adapted from ref. [53]). Weighted UniFrac distances [54] were computed on an abundance table averaged from 100 rarefactions at 899 sequences depth. Differential abundance of OTUs between treatments for each soil layer was assessed using DESeq2 negative-binomial Wald test on non-rarefied reads [55, 56], and indications of the ecology of the most abundant OTUs (>0.5% of rarefied reads) affected by the deep-thaw manipulation were inferred based on their taxonomy and the ecology of their closest relatives (BLAST [57], SI Table S2 [58–72]).

### Statistical analyses

All statistical analyses were carried out using R v3.4.1 [73], and the script used to produce figures and tables is available at https://bitbucket.org/smonteux/monteux_deep_thaw. Effects of the deep-thaw treatment and soil depth on potential respiration at 11 °C, *Q*_10_, and soil abiotic variables (OMC, pH, NH_4_^+^, and soil moisture) were assessed using analysis of variance with linear mixed-effects models (*nlme* package [74]), using treatment and depth as fixed factors, and core as a random factor, followed by pairwise contrasts when appropriate (*lsmeans* package [75]). NH_4_^+^ and both bulk and intrinsic potential respiration were log-transformed, and OMC was square-root-transformed to improve normality of residuals.

The effects of depth and treatment on BCS were assessed by testing for deviation of each OTU against a negative-binomial distribution with generalized linear models (*manyglm(), mvabund* package [76]), which confounds location and dispersion effects less than distance-based approaches (e.g., PERMANOVA [77]). A principal coordinates analysis (PCoA) ordination of weighted UniFrac distances was computed from the averaged abundance table and associations with root density and soil variables were tested using *envfit()* (*vegan* package [78]).

To explore relationships between potential respiration on one hand, and soil chemistry, root density, and BCS (first PCoA axis, henceforth bacterial PCo1) on the other hand, we used multiple linear regressions. Soil chemistry variables showed multi-collinearity, therefore we used principal component analysis to summarize variability in soil chemistry, with all input variables scaled and centered. The first principal component (soil PC1) was mostly driven by positive OMC and soil moisture (SI Fig. S2) and was used as a proxy for soil chemistry in the multiple regressions. Regression models were fit separately for bulk and intrinsic potential respiration, using soil PC1 only, or adding bacterial PCo1 and/or root density (scaled and centered) as predictors. To investigate how predictors of potential respiration differed between control and deep-thaw soils, we included treatment and its interactions with the other independent predictors in all models. We further fitted models with bacterial PCo1 alone to compare the relative importance of soil PC1 and bacterial PCo1. For both bulk and intrinsic potential respiration, we compared regression models based on their second-order Akaike's Information Criterion (AICc, *AICcmodavg* package [79]).

## Results

### Soil chemistry and root density

Soil moisture, organic matter, and carbon content were unaffected by the deep-thaw manipulation and were lower in the permafrost layer relative to the other two soil layers for both treatments. pH was higher in the permafrost than in the active layer in control soils but not in deep-thaw, while NH_4_^+^ was unaffected by depth and treatment (Table 1, SI Table S3, SI Fig. S3). Roots cannot grow into frozen soil, therefore root density distribution reflected whether the soil was seasonally thawed or not (SI Fig. S4). Most of the root biomass belonged to sedges and shrubs.

**Table 1:** Effects of decadal in situ deep-thaw and depth on soil abiotic variables, respiration rates, and bacterial community structure (BCS); association between environmental variables and BCS

Soil chemistry and respiration (ANOVA)	Decadal deep thaw (treatment)		Depth		Treatment:depth	
	*F*	*p*	*F*	*p*	*F*	*p*
Square-root organic matter content (OMC)	2.839	0.123	24.972	**<10** ^**−4**^	0.320	0.730
pH	1.365	0.270	4.847	**0.019**	6.797	**0.006**
Log ammonium	0.074	0.792	0.936	0.409	3.260	0.060
Moisture (gravimetric)	2.012	0.187	7.795	**0.003**	1.126	0.344
Log bulk respiration (per g dry weight)	5.254	**0.045**	19.764	**<10** ^−4^	0.658	0.529
Log intrinsic respiration (per g OM)	0.232	0.641	0.417	0.665	3.920	**0.037**
*Q*_10_ (11–21 °C)	3.360	0.097	5.770	**0.011**	4.101	**0.032**
Bacterial community structure (BCS)	Dev	*p*	Dev	*p*	Dev	*p*
*ManyGLM*	1579	**0.006**	3711	**0.001**	1101	**0.001**
Association with BCS *(envfit*)	PCo1	PCo2	*R* ^2^	*p*		
Organic matter content (OMC)	−0.990	−0.144	0.231	**0.014**		
pH	0.819	0.573	0.111	0.147		
Ammonium	−0.113	0.994	0.317	**0.004**		
Moisture (gravimetric)	−1.000	0.005	0.234	**0.015**		
Root density	−0.804	−0.595	0.233	**0.022**		

Bold text denotes significant *p*-values (*p* < 0.05)

### Bacterial community structure

**Fig. 1:**
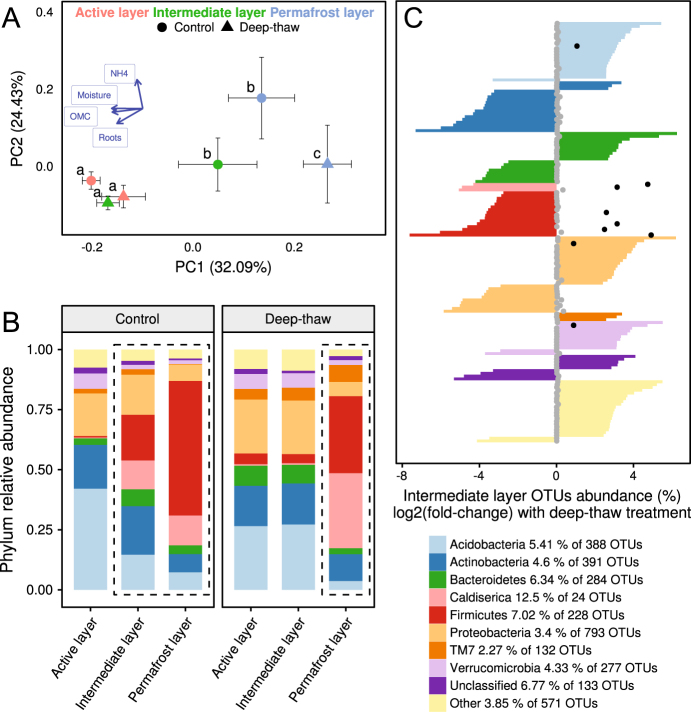
Bacterial community structure based on 16S rRNA V3 region. **a** Principal coordinates analysis of weighted UniFrac distances; markers and error bars represent mean and standard error, respectively, of coordinates on the ordination axes for each depth × treatment combination (*n* = 6, except intermediate layer–deep thaw where *n* = 5); different letters denote significantly different communities (*manyglm* post hoc, see SI Table S4). **b** Mean relative abundance of phyla in each depth × treatment combination; dashed rectangles enclose permanently frozen samples. **c** Phylum distribution, abundance, and abundance change of the 149 OTUs that are different in the intermediate layer between the deep-thaw and control treatments (DEseq2 NB Wald test). Each bar represents the log2 of the fold change between control and deep-thaw samples for one OTU; dots represent the relative abundance of the OTU in the entire rarefied dataset, black dots indicate abundant OTUs (>0.5%, SI Table S2); number of OTUs in the legend is for the intermediate layer, while the percentages show what portion of those is in (**c**)

BCS varied strongly with depth and was also affected by the decadal in situ deep-thaw manipulation, but this effect differed between soil layers (Table 1, Fig. 1). Overall, BCS did not differ across the three seasonally thawed soil layers (active layer in both treatments, intermediate layer in deep-thaw treatment; Fig. 1a). The three permanently frozen soil layers (intermediate layer in control treatment and permafrost layer in both treatments) differed from the seasonally thawed cluster (Fig. 1a, SI Table S4). Seasonal thawing in the intermediate layer thus led to a BCS undistinguishable from the active layer within a decade. This shift from perennially frozen to seasonally thawed BCS was characterized at the phylum level by large decreases in Firmicutes and Caldiserica, and an increase in Acidobacteria relative abundances (Fig. 1b). Soil moisture content, OMC, and root density were mainly associated with PCo1, reflecting the strong effects of depth and thawing, while NH_4_^+^ was associated with PCo2.

Although in the active layer the BCS did not significantly differ between treatments, 321 OTUs showed significant changes (SI Fig S5). Eight relatively abundant OTUs (>0.5% of the rarefied reads) were more abundant in deep-thaw plots, of which four were putative aerobes or micro-aerobes and two were putative anaerobes (SI Table S2).

In the intermediate layer, 149 OTUs changed relative abundance with deep thaw, of which 9 were particularly abundant (Fig. 1c). Among these, the relative abundance of 6 obligate and putative anaerobes decreased, while putative aerobes had an increased relative abundance under deep thaw (SI Table S2).

In the permafrost layer, 48 OTUs changed relative abundance with deep thaw (SI Fig S5), even though no phase change was observed, of which 1 unclassified OTU was abundant and its relative abundance increased in the deep-thaw treatment (SI Table S2).

### Potential aerobic respiration

**Fig. 2:**
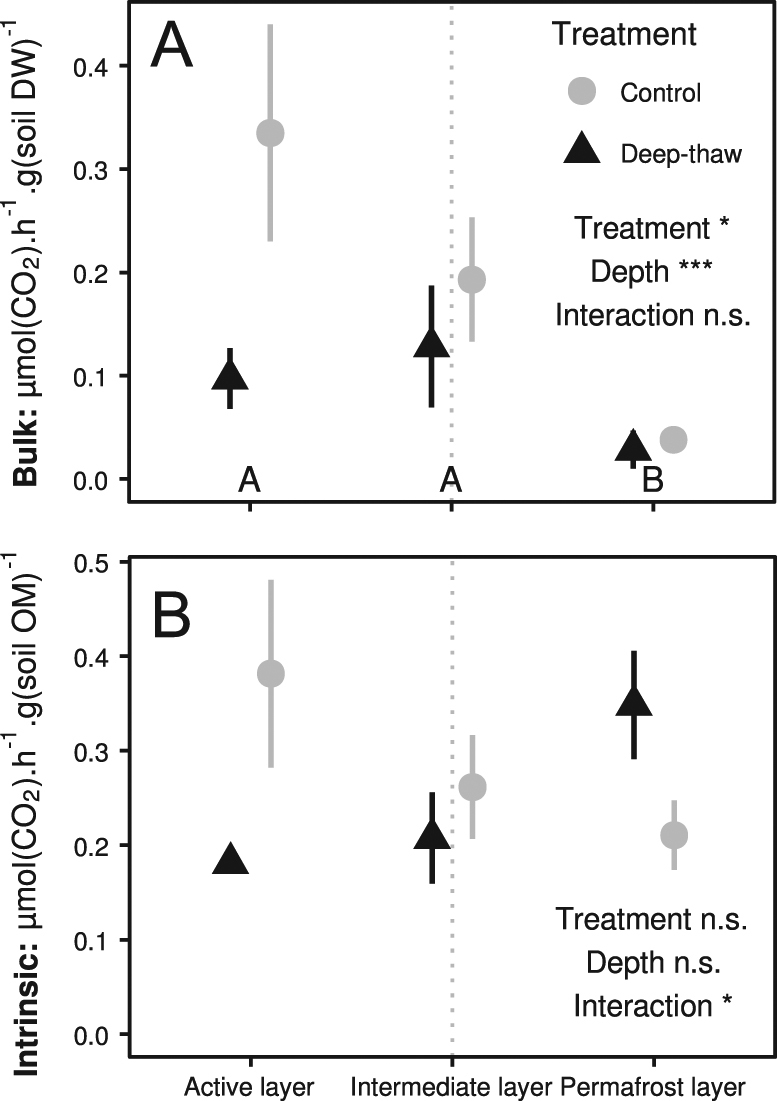
Potential aerobic respiration of soils from different depths in control and decadal in situ deep-thaw plots during laboratory incubation at 11 °C, measured for 1 week after 1 month pre-incubation, expressed per gram soil dry weight (**a**) and per gram soil organic matter (**b**); samples to the right of the dotted line are from permanently frozen soil layers; means ± SE (*n* = 6). Asterisks denote significant effects (*P < 0.05; ***P < 0.001; n.s. non significant), letters denote significant differences between depths

Bulk potential respiration rates (per g soil dry weight) as measured in aerobic soil incubations were 85% (55–95% ± SE, *P* < 0.001) lower in the permafrost layer than in the overlying layers, and were further reduced by 61% (41–74%, *P* = 0.045) in the deep-thaw treatment across all soil layers (no depth × treatment interaction; Table 1, Fig. 2a). In contrast there was no overall effect of depth or treatment on intrinsic potential respiration (per g organic matter dry weight), but a significant depth × treatment interaction (*P* = 0.037, Table 1, Fig. 2b), reflecting lower potential respiration by 45% (26–59%) in the active, 18% (−10–39%) in the intermediate but higher by 74% (30–134%) in the permafrost layer, in soils from the deep-thaw plots than from the control plots. Similarly, there was no main effect of treatment or depth on the temperature sensitivity of respiration (*Q*_10_, calculated between 11 and 21 °C) but a significant depth × treatment interaction indicating that *Q*_10_ varied between the intermediate and permafrost layer primarily in the deep-thaw plots (Table 1, SI Fig. S6, SI Table S5).

### Abiotic and biotic drivers of potential respiration

**Fig. 3:**
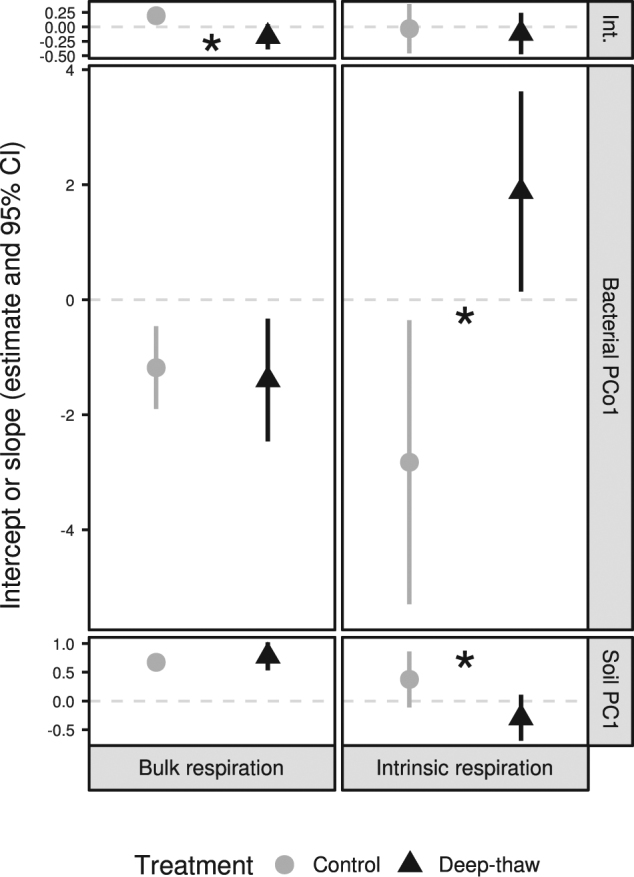
Intercepts and slopes (95% CI) of the best-fitting multiple linear regressions of respiration against soil chemistry (soil PC1) and bacterial community structure (bacterial PCo1). Asterisks denote significant differences between treatments (*P* < 0.05). Int. intercept

Including the experimental treatment alongside soil chemistry (soil PC1), BCS (bacterial PCo1), and/or root density improved all regression models for both bulk and intrinsic potential respiration (SI Table S6). Bulk respiration models best fit the data when adding treatment without interactions (treatment effect on intercept) while for intrinsic respiration all interactions between treatment and other predictors significantly improved model fits (effect on slopes and intercept; Fig. 3, SI Table S6). Although soil PC1 slopes did not differ from zero, the significantly different slopes between control and deep-thaw samples for both bacterial PCo1 and soil PC1 reflected the depth × treatment interaction on intrinsic respiration previously described (Figs. 2b, 3, and Table 2).

**Table 2:** Multiple regressions between potential soil respiration at 11 °C, and soil chemistry, bacterial community structure, and root density in soils from different depths in control and decadal in situ deep-thaw plots

				Control samples		Deep-thaw samples		Deep-thaw (treatment) effect	
	AICc	*R* ^2^	Variable	Slope (±SE)	*p*	Slope (±SE)	*p*	Intercept (±SE)	*p*
Predictors of bulk respiration (per g DW)									
Soil × treatment	48.30	0.848	Soil PC1	0.823 ± 0.074	**<** **0.001**	−0.892 ± 0.126	**<** **0.001**	−0.300 ± 0.146	0.066
(Soil + bacteria) × treatment	39.81	0.901	Soil PC1	0.687 ± 0.075	**<** **0.001**	0.755 ± 0.119	**<** **0.001**	−0.376 ± 0.123	**0.012**
			Bacterial PCo1	−1.176 ± 0.381	**0.011**	−1.344 ± 0.514	**0.028**		
(Soil + root density) × treatment	49.15	0.871	Soil PC1	0.776 ± 0.080	**<** **0.001**	0.896 ± 0.120	**<** **0.001**	−0.451 ± 0.168	**0.023**
			Root density	0.341 ± 0.235	0.177	0.140 ± 0.086	0.138		
(Root density + soil + bacteria) × treatment	46.84	0.903	Soil PC1	0.687 ± 0.078	**<** **0.001**	0.775 ± 0.127	**<** **0.001**	−0.392 ± 0.156	**0.031**
			Root density	0.011 ± 0.243	0.964	0.052 ± 0.092	0.589		
			Bacterial PCo1	−1.165 ± 0.472	**0.036**	−1.169 ± 0.611	0.092		
Bacteria × treatment	86.82	0.538	Bacterial PCo1	−3.079 ± 0.772	**0.002**	−2.830 ± 0.811	**0.006**	−0.778 ± 0.243	**0.009**
Predictors of intrinsic respiration (per g SOM)									
Soil × treatment	97.65	0.367	Soil PC1	0.716 ± 0.226	**0.009**	−0.445 ± 0.184	**0.036**	−0.161 ± 0.295	0.597
(Soil + bacteria) × treatment	93.33	0.539	Soil PC1	0.402 ± 0.240	0.125	−0.268 ± 0.182	0.176	−0.195 ± 0.265	0.477
			Bacterial PCo1	−2.826 ± 1.198	**0.040**	1.744 ± 0.787	0.054		
(Soil + root density) × treatment	101.93	0.408	Soil PC1	0.657 ± 0.247	**0.024**	−0.450 ± 0.178	**0.032**	−0.260 ± 0.358	0.484
			Root density	0.485 ± 0.723	0.518	−0.181 ± 0.128	0.192		
(Root density + soil + bacteria) × treatment	100.16	0.549	Soil PC1	0.405 ± 0.245	0.133	−0.292 ± 0.195	0.172	0.054 ± 0.333	0.875
			Root density	−0.466 ± 0.766	0.558	−0.065 ± 0.141	0.656		
			Bacterial PCo1	−3.314 ± 1.463	**0.050**	1.524 ± 0.939	0.144		
Bacteria × treatment	92.45	0.456	Bacterial PCo1	−3.941 ± 1.051	**0.003**	2.251 ± 0.734	**0.012**	−0.228 ± 0.263	0.407

*Soil* soil PC1, *Bacteria* bacterial PCo1

All variables are scaled and centered before analyses; bold text denotes significant *p*-values (*p* < 0.05)

Models combining BCS and soil chemistry best described both bulk and intrinsic potential respiration, with decreases in AICc compared to models based on soil chemistry only of 8.43 and 4.33, respectively (Table 2). When including bacterial PCo1 only, the fit for bulk respiration was worse, but for intrinsic respiration better than in models with soil PC1 alone (ΔAICc −5.2, Δ*R*^2^ +9%). The model using bacterial PCo1 only for intrinsic respiration was similarly well-fitting as the model including both soil PC1 and bacterial PCo1 (ΔAICc −0.88, Δ*R*^2^ −8%, likelihood-ratio test *P* = 0.059, Table 2).

## Discussion

In line with our hypothesis (a), the response of the BCS to the deep-thaw treatment was strongest in the intermediate layer where the permafrost changed from permanently frozen to seasonally thawed (Fig. 1). The convergence of the deep-thaw intermediate layer BCS with the active layer communities supports previous findings of BCS differing between active layer and permafrost in the field [26, 81], and of convergence toward BCS found in the active layer upon permafrost thawing in vitro [28]. Furthermore, although the convergence might also partly stem from the differential growth of certain endogenous taxa, the strikingly similar communities among seasonally thawed soils suggests that overlying soil microorganisms migrated to newly thawed soil. One previous study has compared BCS at the same depth in seasonally thawed and permafrost soil in sites with permafrost degradation following fire, but there, fire and thaw effects were confounded [80]. Our results therefore provide the first in situ experimental evidence of the BCS response to permafrost thaw. Long-term freezing constraints can be a more important determinant of BCS than soil depth per se in permafrost soils, and once those constraints are relieved the overlying microorganisms seem to migrate, determining the resulting BCS.

Although the deep-thaw experiment increased soil moisture in the upper soil layers [41], this effect did not extend to the deeper soil layers where we took our samples. Instead, increases in relative abundances of putative aerobic and anaerobic OTUs between treatments suggest that oxygen might have reached deeper soil layers in the deep-thaw plots, where *E. vaginatum* is more abundant. *Eriophorum* roots harbor aerenchymae, likely making deep soil more oxic (SI Fig. S4 & S7 [13, 17, 81]).

High relative abundances (up to 60%) of the phylum Caldiserica were found in the permanently frozen soil layers (Fig. 1b). This poorly known phylum has one cultured representative (thermophilic *Caldisericum exile* [82]), and has been found in other extreme environments (e.g. [83]). Wurzbacher et al. [84] recently showed that enigmatic microbial phyla dominate permafrost thaw ponds. The high relative abundances we observed suggest that intact permafrost, at least in our study system, could be used to investigate Caldiserica ecology, through culturing and -omics approaches.

As hypothesized (b), soils from the active layer in the deep-thaw plots had lower potential respiration rates than their control counterparts (Fig. 2). In contrast, higher ecosystem respiration in deep-thaw plots was observed in the first years of this experiment [40], when the increase in ALT, and thus in thawed SOM, was still limited (~6 cm). We suggest that enhanced SOM decomposition and processing due to higher temperatures and prolonged thaw period over a decade of manipulation have increasingly depleted the labile C-pool, as Semenchuk et al. [9] suggested in a similar experiment.

In the intermediate layer, our hypothesis (b) of higher potential respiration because of increased microbial functional diversity was refuted: instead, both bulk and intrinsic potential respiration appeared lower in the deep-thaw plots. Upon permafrost thawing, fast metabolic responses facilitate SOM decomposition [28, 85], which could have increased carbon losses in the short term. Short periods of oxygenation, e.g., through *E. vaginatum* aerenchymae, can have lasting consequences on decomposition, by promoting phenol oxidase activity [18, 86], and seem plausible considering the observed changes in aerobic and anaerobic OTU relative abundances. Moreover, similar temperature effects as described above could result in the lower respiration rates observed after 10 years, through labile C-pool depletion, although we presumed the shorter thaw period in intermediate layers would delay such effects. This is further supported by higher *Q*_10_ values in the deep-thaw intermediate layer, suggesting increased chemical recalcitrance (SI Fig. S6 [87]). While labile C-pool depletion has been previously suggested to explain decreased soil respiration [9, 88] and would fit our observations, integrated soil respiration measurements over a long-term experiment coupled with organic matter profiling would be necessary to confirm this interpretation.

Deep thaw had contrasting effects in the permafrost layer, with decreased bulk respiration and increased intrinsic respiration. Deep thaw may raise temperatures in still-frozen soil layers at most by <1 °C [74]. Combined with the observed higher rate of intrinsic respiration, it therefore seems unlikely that the aforementioned labile carbon depletion would also apply in the frozen soil. Alternatively, the different BCS in deep-thaw and control permafrost layers (Fig. 1a) might be functionally distinct, potentially leading to the observed differences in respiration. However, we hesitate to propose this as the single underlying mechanism, since the only abundant OTU affected by the manipulation provides no functional insights due to its high divergence from all cultured organisms (OTU_6022, SI Table S2). Further, although the treatment-affected OTUs may reflect a temperature effect on BCS in the permafrost layer, their relatively low number could also indicate spatial sampling effects exacerbated by the low population sizes in permafrost soils.

Our hypothesis (c), according to which including biota improves soil chemistry-based predictions of potential respiration, was supported for both bulk and intrinsic respiration: including BCS resulted in better-fitting models. Moreover, the significant interactions of soil chemistry and BCS with treatment, as well as the opposite directions of the slopes between treatments, strongly suggest that the drivers of intrinsic respiration fundamentally changed under decadal deep thaw (intrinsic respiration increased with, e.g., higher OMC in control soils but decreased in deep-thaw soils). Although all models including BCS showed a significantly better fit than soil-only models, bulk respiration rates were almost equally well explained by soil chemistry alone. In contrast, BCS alone explained intrinsic respiration rates better than soil chemistry, implying that after accounting for different OMC, BCS can explain variation in respiration better than soil chemistry. We cannot rule out that microbial biomass or other soil chemistry measures, e.g., recalcitrance of SOM [89, 90], may explain additional variation in respiration. Further, if BCS and respiration share these unmeasured variables as common drivers, some of the variation we currently attribute to BCS could in fact be due to other factors. 16S-based community profiles, as used here, give only limited information about functional potential. However, our results are in line with recent evidence suggesting that bacterial taxonomic composition can explain a unique portion of variation in respiration (agricultural temperate soil [91]), and partly determines soil carbon dynamics [92], particularly in organic soils where microbial activity is less constrained [94–95]. While the underlying mechanisms in terms of community functionality are unclear, our results suggest that measuring BCS may improve predictions of the permafrost carbon feedback.

In contrast with our hypothesis (c), including root density did not improve models of bulk or intrinsic potential respiration. Root density might affect soil respiration as measured in incubations through legacy effects of either rhizosphere priming, depleting the SOM pool, or alterations of BCS in the rhizosphere. The limited contribution of root density to our models is in agreement with relatively small (4%) priming effects observed in peatlands [96], while the rhizosphere–BCS effects may already be accounted for by the overall changed BCS, thus explaining only some of the information that BCS contributes. In addition to such legacy effects possibly observed in incubations, higher root density might also affect soil respiration directly in situ, e.g., by stimulating rhizosphere microbial activity or by increasing SOM turnover without affecting the size of the SOM pool. Using different experimental designs, such as root exclosures, might allow to disentangle direct and legacy effects of root colonization effects from other consequences of thawing [97].

Our in situ approach confirmed the large changes in BCS after experimental permafrost thaw previously observed in incubations [28, 85], and further suggests that colonization by active layer bacteria might determine the fate of BCS in thawing permafrost. While root density appeared uninformative in predicting respiration, the strong linkages between BCS and potential respiration challenge the view that in soils BCS is unimportant for C-cycling, at least in our system, particularly after accounting for variations in SOM content. This BCS–C-cycling link, and underlying functional implications, should be investigated in other permafrost-affected environments, as it could imply that current predictions for SOM decomposition in thawing permafrost omit an important component. Altogether, our results show that permafrost thaw indirectly affects SOM decomposition through large effects on its drivers, such as BCS, which might prove important in understanding and predicting the permafrost carbon feedback.

The data supporting the findings of this study are available in figshare with the identifier 10.6084/m9.figshare.5977471; the sequence data have been deposited in Sequence Read Archive with accession numbers SAMN07445364–SAMN07445398 and SAMN07445422; the entire bioinformatics and statistical analysis pipeline is available at https://bitbucket.org/smonteux/monteux_deep_thaw/.

## Electronic supplementary material

Supplementary Figures

Supplementary Tables
