# Supplementary material for: Long-term in situ permafrost thaw effects on bacterial communities and potential aerobic respiration
Source: ISME J. 2018 Jun 6;12(9):2129–41. doi: 10.1038/s41396-018-0176-z (PMC6092332; doi:10.1038/s41396-018-0176-z)

Supplementary Figure S1: DNA content of (A) DNA extracts and (B) soil samples. Soil DNA content was calculated from DNA concentration of the extract and dry weight of the soil used for extraction; means ± SE (n=6)

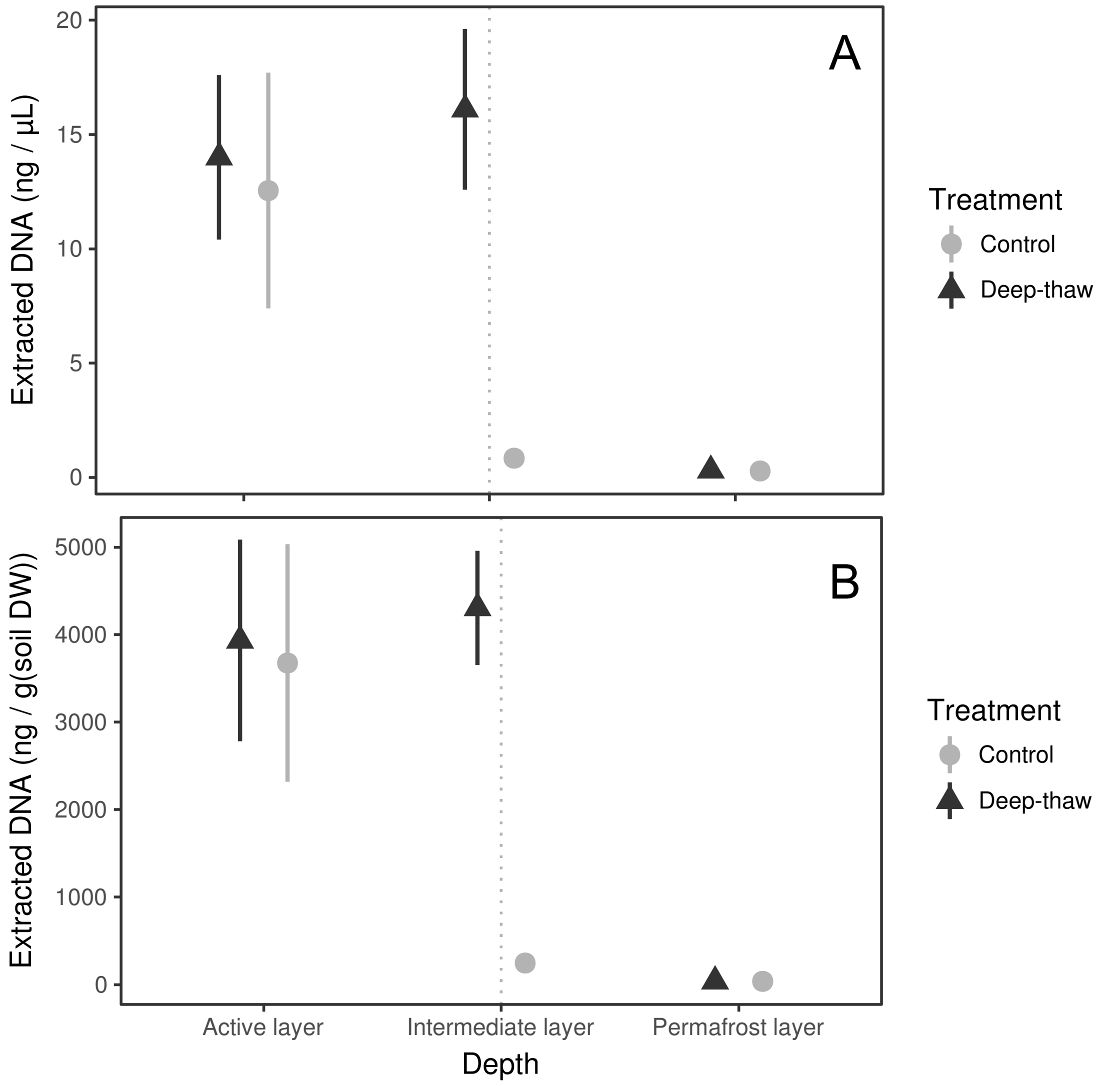

Supplementary Figure S2: Principal components analysis of soil chemistry variables, principal components 1 and 2; arrows indicate the soil chemistry variables (OMC = Organic matter content), each number is a sample.

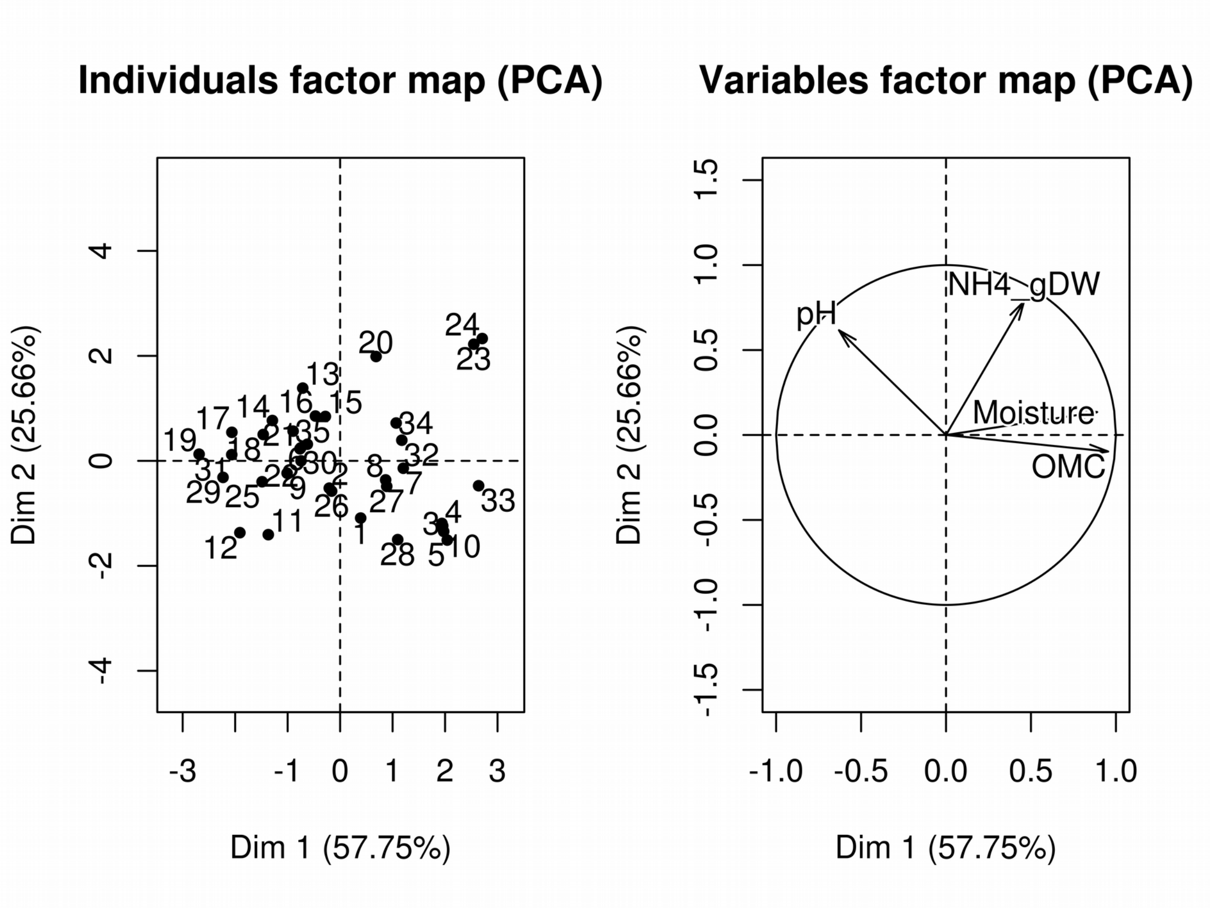

Supplementary Figure S3: Abiotic soil variables across soil depths and in response to decadal *in situ* deep-thaw; means ± SE (n=6, except Carbon content where n=3); upper-case letters indicate differences between depths; lower-case letters indicate differences between depth x treatment combinations, see SI Table S3.

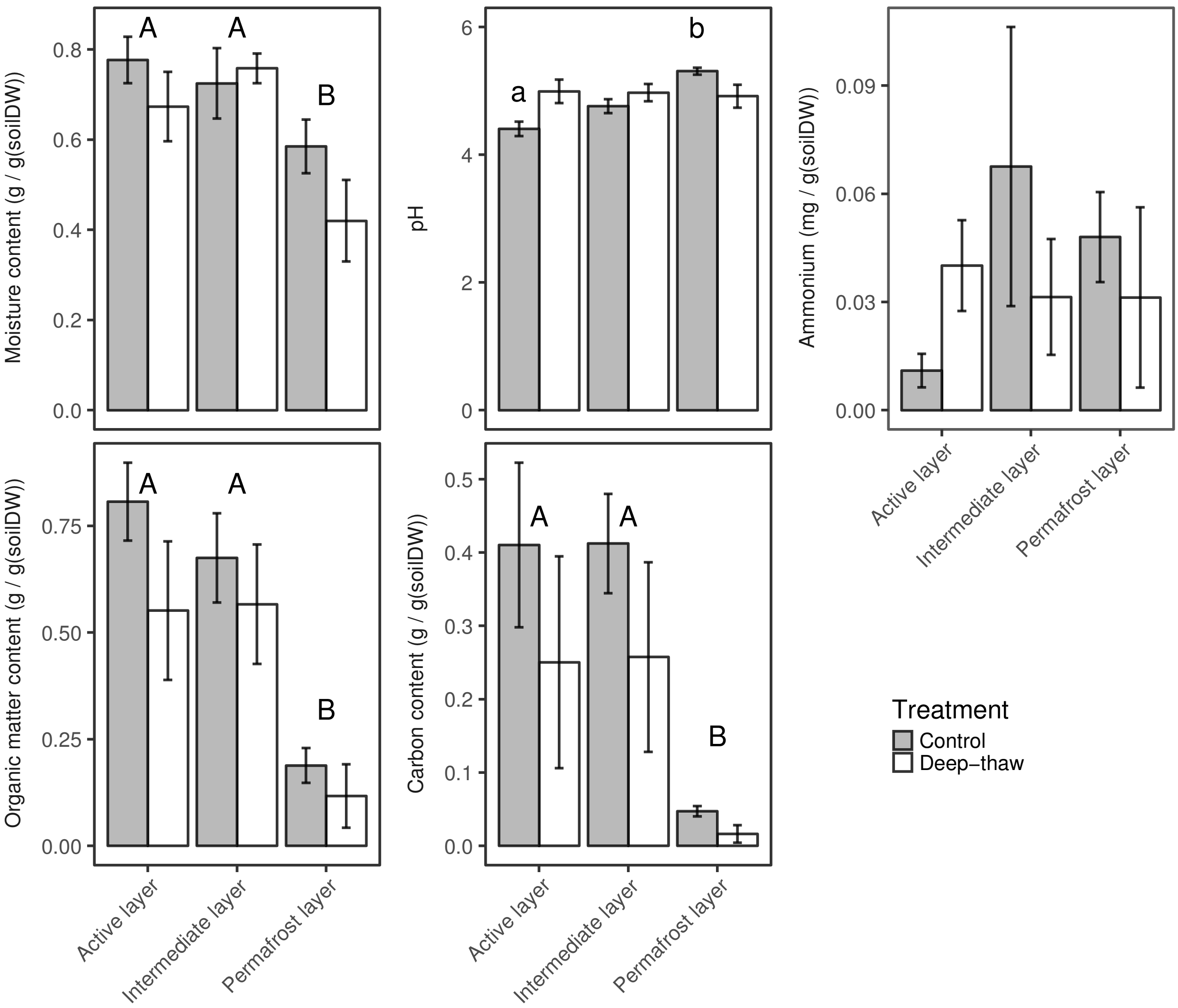

Supplementary Figure S4: Root density by plant functional type, in different soil depths and in response to decadal *in situ* deep-thaw; means ± SE (n=6).

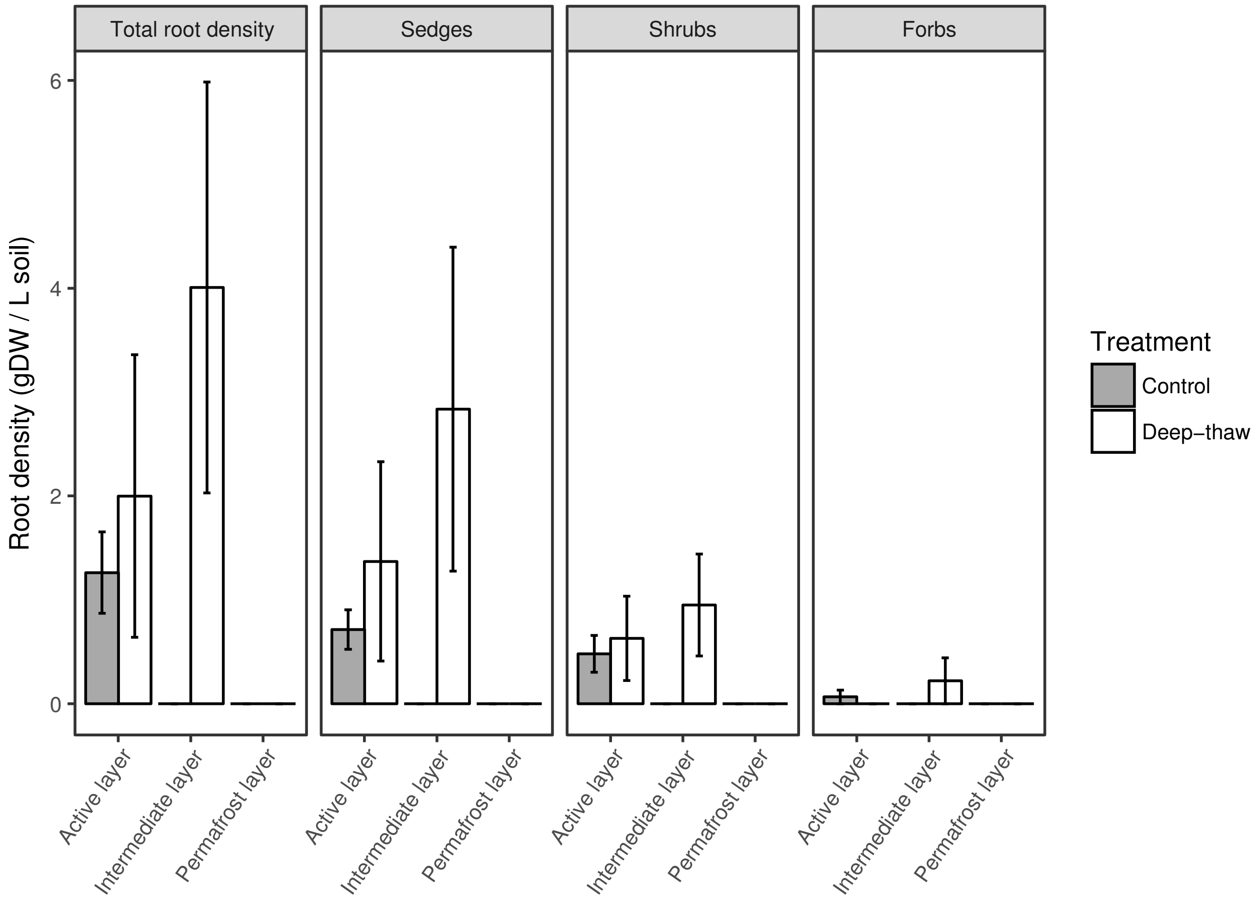

Supplementary Figure S5: Phylum-distribution, relative abundance and abundance change of the 321 and 48 OTUs that are different in the active and permafrost layer, respectively, between the deep-thaw and control treatments (DEseq2 NB Wald test). Each bar represents the log2 of the fold-change between control and deep-thaw samples for one OTU; dots represent the relative abundance of the OTU in the entire rarefied dataset, black dots indicate abundant OTUs (>0.5%); number of OTUs in the legend is the total in the respective soil layer in the rarefied dataset, while the percentages show what portion of those is present in the display

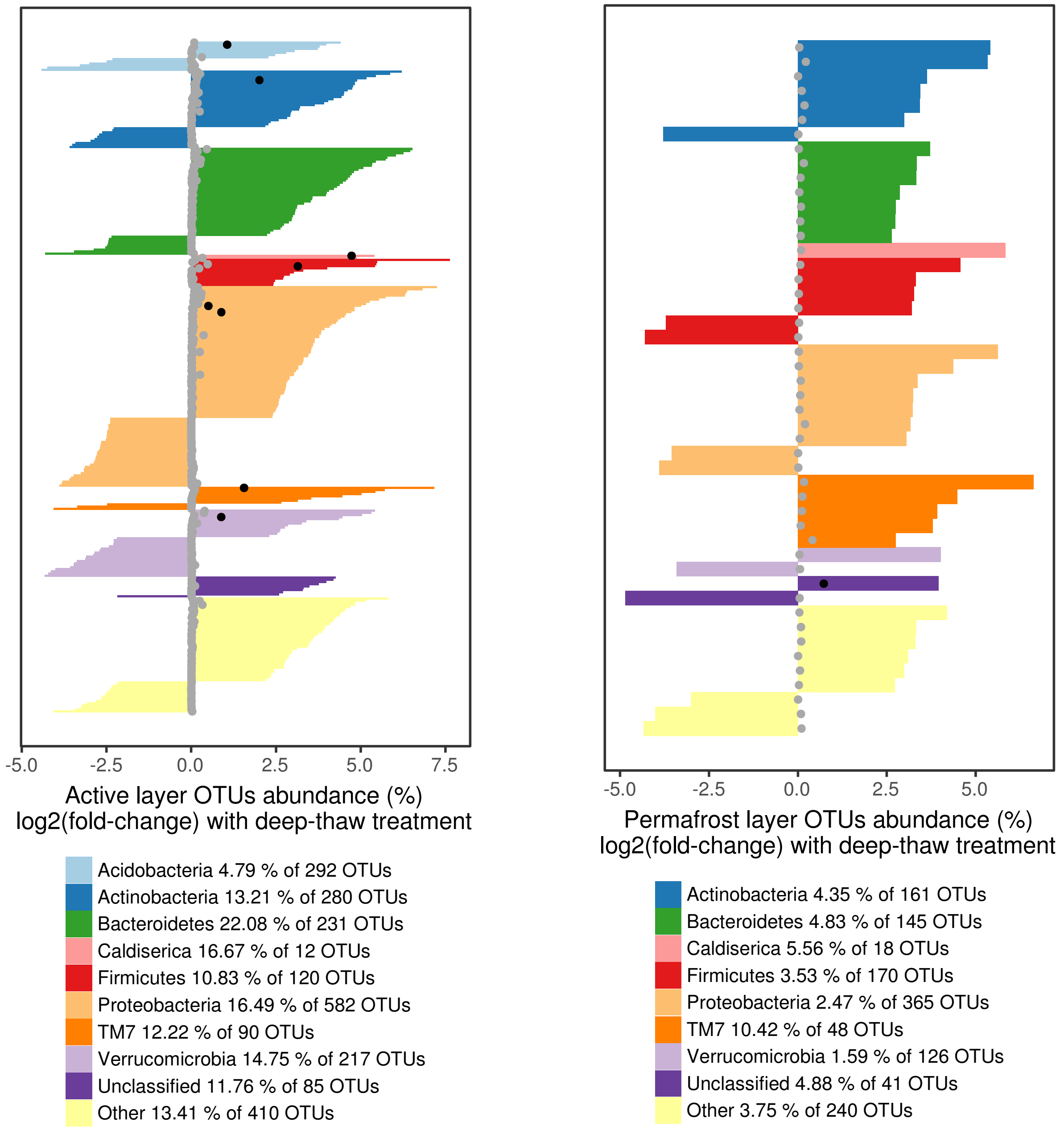

Supplementary Figure S6: Temperature sensitivity (Q_{10}) of potential respiration from soils from different depths in control and decadal *in situ* deep-thaw plots. Ratio between respiration at 21ºC and 11ºC; means ± SE (n=6); different letters indicate significant differences between depths (SI Table S4).

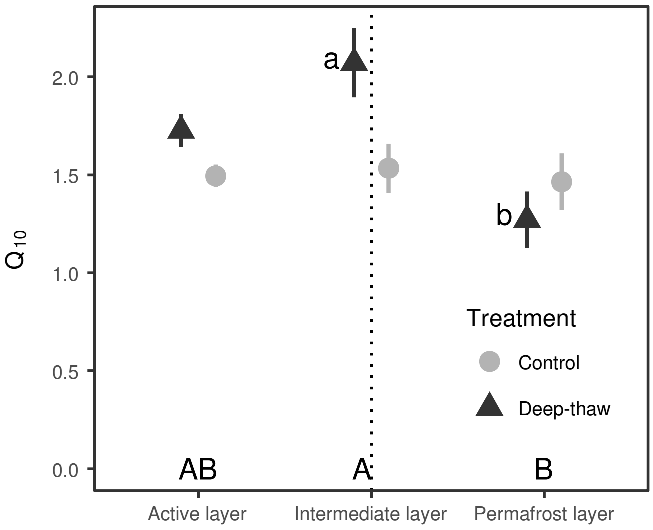

Supplementary Figure S7: Plant functional types cover in control and decadal *in situ* deep-thaw plots visually estimated on 0.5x0.5m quadrats, means ± SE (n=6); asterisks denote significant ($p<0.05$) differences between treatments (ANOVA)

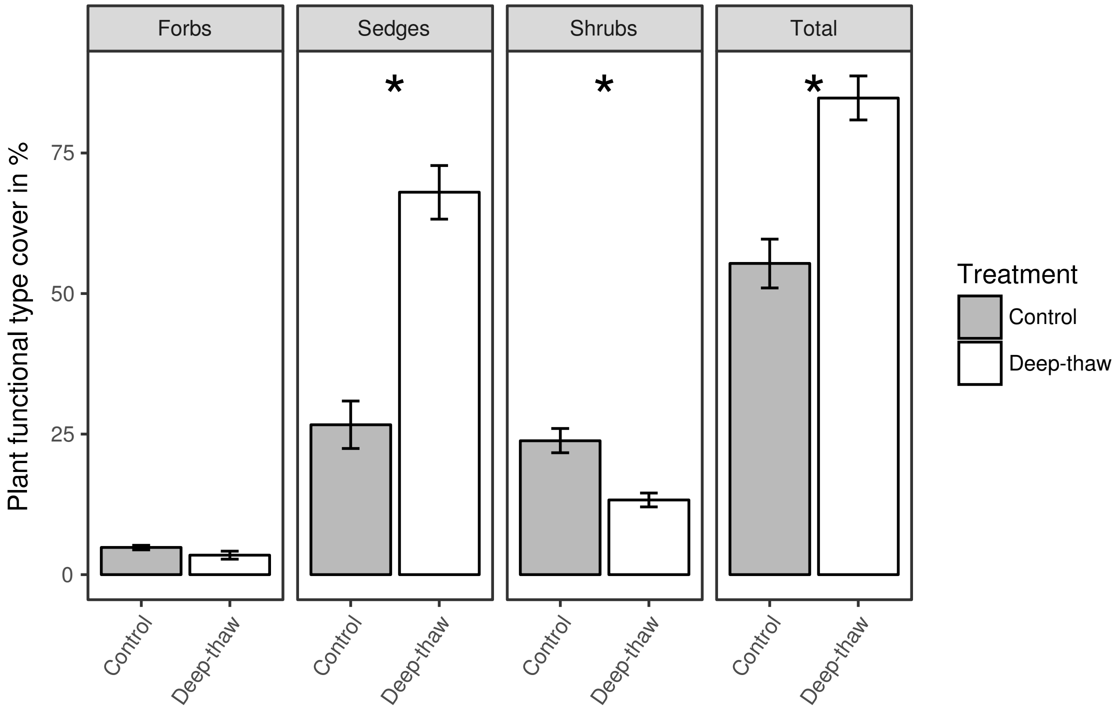

Supplement: Supplementary file 1 — Supplementary Figures [file 41396_2018_176_MOESM1_ESM.pdf]
