# Supplementary material for: Long-term in situ permafrost thaw effects on bacterial communities and potential aerobic respiration
Source: ISME J. 2018 Jun 6;12(9):2129–41. doi: 10.1038/s41396-018-0176-z (PMC6092332; doi:10.1038/s41396-018-0176-z)
Supplement: Supplementary file 2 — Supplementary Tables [file 41396_2018_176_MOESM2_ESM.pdf]

Supplementary Table S1: PCR conditions and primers sequences

PCR-reaction mix

Phusion High-Fidelity PCR MasterMix	12.5μL
Nuclease-free water	8μL
Forward primer (V3_F)	1.25μL at 10μM
Reverse primer (V3_R, 96 different indexes)	1.25μL at 10μM
DNA template	2μL at ca. 5ng/μL

PCR conditions

Initial denaturation		98°C	1min
25 cycles of	Denaturation	98°C	10s
	Annealing	50°C	30s
	Elongation	72°C	30s
Final elongation		72°C	7min

Primers

V3_F (341F)	aatgatacggcgaccaccgagatctacactctttccctacacgacgctcttccgatctCCTACGGGAGGCAGCAG
V3_R (518R)	caagcagaagacggcatacgagat**BBBBBB**gtgactggagttcagacgtgtgctcttccgatctATTACCGCGGCTGCTGG

Lower-case letters and underlined letters are Illumina adapters and sequencing primers, respectively; upper-case letters are PCR-primers 341F and 518R, bold uppercase are the 6 bases used for indexing (96 different indexes, as listed in Bartram et al. 2011, AEM doi:10.1128/AEM.02772-10)

Supplementary Table S2: Representative sequences, taxonomy, closest GenBank relatives and references for ecology of abundant OTUs; abundances are expressed in % of total reads in the rarefied dataset, colored text in each layer indicates the effect of deep-thaw on the relative abundance (DESEQ negative-binomial Wald test)
When taxonomy assignment conflicted between RDP and BLAST, the RDP taxonomy (based on GreenGenes 13_8) was preferred;
Bold GenBank ID indicate the sequence comes from a cultured strain

**OTU_2**	**Relative abundance**	**Active layer**	**Intermediate layer**	**Permafrost layer**	**Assignment**	**Phylum**	**Class**	**Order**	**Family**	**Genus**	**Species**	**GenBank ID**	**Growth**	**Reference**	**DOI**
					RDP	Firmicutes	Clostridia	Clostridiales	Clostridiaceae	Clostridium	sp.		Anaerobic	Rainey et al., 2015	10.1002/9781118960608.gbm00619
	4.91%		Decrease		Blast	Firmicutes	Clostridia	Clostridiales	Clostridiaceae	Clostridium	aestuari	**NR_043569.1**	Anaerobic	Kim et al. 2007	10.1099/ijs.0.64428-0
						TGGGGAATATTGCGCAATGGGGGAAACCCTGACGCAGCAACGCCGCGTGGGTGATGAAGGTCTTCGGATTGTAAAGCCCTGTCTTTTGGGACGATAATGACGGTACCAAAGGAGGAAGCCACGGCTAACTACGTG									
**OTU_4**	**Relative abundance**	**Active layer**	**Intermediate layer**	**Permafrost layer**	**Assignment**	**Phylum**	**Class**	**Order**	**Family**	**Genus**	**Species**	**GenBank ID**	**Growth**	**Reference**	**DOI**
					RDP	Firmicutes	Clostridia	Clostridiales	Clostridiaceae	Clostridium	ljungdahlii		Anaerobic	Tanner et al., 1993	10.1099/00207713-43-2-232
	3.14%	Increase	Decrease		Blast	Firmicutes	Clostridia	Clostridiales	Clostridiaceae	Clostridium	carboxidivorans	**KU316945.1**	Anaerobic	Liou et al., 2005	10.1099/ijs.0.63482-0
					Blast	Firmicutes	Clostridia	Clostridiales	Clostridiaceae	Clostridium	drakei	**KR997833.1**	Anaerobic	Liou et al., 2005	10.1099/ijs.0.63482-0
						TGGGGAATATTGCACAATGGGCGAAAGCCTGATGCAGCAACGCCGCGTGAGTGATGAAGGCCTTCGGGTTGTAAAGCTCTGTCTTTGGGGACGATAATGACGGTACCCAAGGAGGAAGCCACGGCTAACTACGTG									
**OTU_5**	**Relative abundance**	**Active layer**	**Intermediate layer**	**Permafrost layer**	**Assignment**	**Phylum**	**Class**	**Order**	**Family**	**Genus**	**Species**	**GenBank ID**	**Growth**	**Reference**	**DOI**
					RDP	Caldiserica	WCHB1-03						Anaerobic	Mori et al., 2009	10.1099/ijs.0.010033-0
	4.73%	Increase	Decrease		Blast	Caldiserica	Caldisericia	Caldisericales	Caldisericaceae	Caldisericum		LT625619.1	Anaerobic	Mori et al., 2009	10.1099/ijs.0.010033-0
					Blast	Firmicutes	Clostridia	Clostridiales	Clostridiaceae	Clostridium		GU000298.1	Anaerobic	Rainey et al., 2015	10.1002/9781118960608.gbm00619
						TGGGGAATCATGGTCAATGGGCGAAAGCCTGAACCTGCGACGCCGCGTGAGTGATGAAGGTCGTAAGATCGTAAAACTCTTTTCAGGGACATTAAAGCTCCGGCTCTAACAGAGCCTGGAGTATGACTATCTCTGGAATAAGCCCCAGCCAACTACGTG									
**OTU_7**	**Relative abundance**	**Active layer**	**Intermediate layer**	**Permafrost layer**	**Assignment**	**Phylum**	**Class**	**Order**	**Family**	**Genus**	**Species**	**GenBank ID**	**Growth**	**Reference**	**DOI**
					RDP	Firmicutes	Clostridia	Clostridiales	Ruminococcaceae	Ethanoligenens	sp.		Anaerobic	Xing et al., 2006	10.1099/ijs.0.63926-0
	2.59%		Decrease		Blast	Firmicutes	Clostridia	Clostridiales	Ruminococcaceae			JX505347.1	Anaerobic	Rainey 2015	10.1002/9781118960608.fbm00136
					Blast	Firmicutes	Clostridia	Clostridiales	Clostridiaceae			**EU815225.1**	Anaerobic	Wiegel 2015	10.1002/9781118960608.fbm00129
						TGGGGGATATTGCACAATGGAGGAAACTCTGATGCAGCGACGCCGCGTGAGGGAAGAAGGTCTTCGGATTGTAAACCTCTGTCTTCAGGGACGAAACAAATGACGGTACCTGAAGAGGAAGCCACGGCTAACTACGTG									
**OTU_8**	**Relative abundance**	**Active layer**	**Intermediate layer**	**Permafrost layer**	**Assignment**	**Phylum**	**Class**	**Order**	**Family**	**Genus**	**Species**	**GenBank ID**	**Growth**	**Reference**	**DOI**
					RDP	Firmicutes	Clostridia	Clostridiales	Clostridiaceae	Clostridium	bowmanii		Anaerobic	Spring et al., 2003	10.1099/ijs.0.02554-0
	2.49%		Decrease		Blast	Firmicutes	Clostridia	Clostridiales	Clostridiaceae	Clostridium		MF360154.1	Anaerobic	Rainey et al., 2015	10.1002/9781118960608.gbm00619
						TGGGGAATATTGCGCAATGGGGGAAACCCTGACGCAGCAACGCCGCGTGAATGATGAAGGCCTTCGGGTTGTAAAGTTCTGTCTTCTGGGACGATAATGACGGTACCAGAGGAGGAAGCCACGGCTAACTACGTG									
**OTU_9**	**Relative abundance**	**Active layer**	**Intermediate layer**	**Permafrost layer**	**Assignment**	**Phylum**	**Class**	**Order**	**Family**	**Genus**	**Species**	**GenBank ID**	**Growth**	**Reference**	**DOI**
					RDP	Acidobacteria	Acidobacteriia	Acidobacteriales	Acidobacteriaceae	candidatus Koribacter	sp.		Aerobic	Ward et al., 2009	10.1128/AEM.02294-08
	1.06%	Increase	Increase		Blast	Acidobacteria						EU849317.1			
					Blast	Acidobacteria	Acidobacteriia	Acidobacteriales	Acidobacteriaceae	candidatus Koribacter	sp.	MF002357.1	Aerobic	Ward et al., 2009	10.1128/AEM.02294-08
						TGGGGAATTTTGCGCAATGGGGGAAACCCTGACGCAGCAACGCCGCGTGGAGGATGAAGCCCCTTGGGGTGTAAACTCCTTTCGATCGGGACGATGATGACGGTACCGGATGAAGAAGCACCGGCTAACTCTGTG									
**OTU_10**	**Relative abundance**	**Active layer**	**Intermediate layer**	**Permafrost layer**	**Assignment**	**Phylum**	**Class**	**Order**	**Family**	**Genus**	**Species**	**GenBank ID**	**Growth**	**Reference**	**DOI**
					RDP	TM7_3		Blgi18							
	1.56%	Increase			Blast	candidatus Saccharobacteria						KX123312.1			
						TGAGGAATCTTCCACAATGGGCGAAAGCCTGATGGAGCGACGCCGCGTGCAGGATGAAGGCCTTAGGGTTGTAAACTGCTTTTATAAGTGAAGAATATGACGGTAACTTATGAATAAGCACCGGCTAACTACGTG									
**OTU_12**	**Relative abundance**	**Active layer**	**Intermediate layer**	**Permafrost layer**	**Assignment**	**Phylum**	**Class**	**Order**	**Family**	**Genus**	**Species**	**GenBank ID**	**Growth**	**Reference**	**DOI**
					RDP	Actinobacteria	Actinobacteria	Micrococcales	Intrasporangiaceae	Phycicoccus	sp.		Aerobic	Zhang et al., 2011	10.1099/ijs.0.020842-0
					Blast	Actinobacteria	Actinobacteria	Micrococcales	Intrasporangiaceae	Tetrasphaera	sp.	KY117522.1	Aerobic	Maszenan et al., 2000	10.1099/00207713-50-2-593
	2.01%	Increase			Blast	Actinobacteria	Actinobacteria	Kineosporiales	Kineosporiaceae	Angustibacter	sp.	LT7191601.1	Aerobic	Tamura et al., 2010	10.1099/ijs.0.019448-0
					Blast	Actinobacteria	Actinobacteria	Micrococcales	Intrasporangiaceae	Phycicoccus	sp.	KP639140.1	Aerobic	Zhang et al., 2011	10.1099/ijs.0.020842-0
						TGGGGAATATTGCACAATGGGCGAAAGCCTGATGCAGCGACGCCGCGTGAGGGATGAAGGCCTTCGGGTTGTAAACCTCTTTCAGCAGGGAAGAAGCGAAAGTGACGGTACCTGCAGAAGAAGCACCGGCTAACTACGTG									
**OTU_13**	**Relative abundance**	**Active layer**	**Intermediate layer**	**Permafrost layer**	**Assignment**	**Phylum**	**Class**	**Order**	**Family**	**Genus**	**Species**	**GenBank ID**	**Growth**	**Reference**	**DOI**
					RDP	Proteobacteria	Beta-	Nitrosomonadales	Gallionellaceae	Gallionella	sp.		Micro-aerobic/aerobic	Emerson et al., 2013	10.1002/9781118960608.gbm00085
					Blast	Proteobacteria	Beta-	Nitrosomonadales	Gallionellaceae	Sideroxydans	sp.	JQ177973.1	Micro-aerobic	Emerson et al., 2013	10.1099/00207713-50-2-593
	0.89%	Increase	Increase		Blast	Proteobacteria	Beta-	Nitrosomonadales	Nitrosomonadaceae	Nitrosospira	sp.	LC053151.1	Aerobic	Head et al., 1993	10.1099/00221287-139-6-1147
					Blast	Proteobacteria	Beta-	Nitrosomonadales	Gallionellaceae	Gallionella	sp.	AB252929.1	Micro-aerobic/aerobic	Emerson et al., 2013	10.1002/9781118960608.gbm00085
						TGGGGAATTTTGGACAATGGGGGCAACCCTGATCCAGCCATGCCGCGTGAGTGAAGAAGGCCTTCGGGTTGTAAAGCTCTTTCAGACGGAAAGAAACGGTCACGGCCAACACCCGTGACTAATGACGGTACCGTCAGAAGAAGCACCGGCTAACTACGTG									
**OTU_14**	**Relative abundance**	**Active layer**	**Intermediate layer**	**Permafrost layer**	**Assignment**	**Phylum**	**Class**	**Order**	**Family**	**Genus**	**Species**	**GenBank ID**	**Growth**	**Reference**	**DOI**
					RDP	Verrucomicrobia	Verrucomicrobiae	Verrucomicrobiales	Subdivion 3 – Ellin515						
	0.89%	Increase	Increase		Blast	Verrucomicrobia	Verrucomicrobiae	Verrucomicrobiales				JQ177487.1			
						TCGAGAATTTTTCACAATGGGCGAAAGCCTGATGGAGCGACGCCGCGTGGGGGATGAATGGCTTCGGCCCGTAAACCCCTGTCATTCGGGATCAATGCGTTTGGGTGAACATCCCAAACGTTGATAGTACCGGAAGAGGAAGGGACGGCTAACTCTGTG									
**OTU_25**	**Relative abundance**	**Active layer**	**Intermediate layer**	**Permafrost layer**	**Assignment**	**Phylum**	**Class**	**Order**	**Family**	**Genus**	**Species**	**GenBank ID**	**Growth**	**Reference**	**DOI**
					RDP	Proteobacteria	Gamma-	Xanthomonadales	Rhodanobacteraceae	Dokdonella	sp.		Aerobic	Yoon et al., 2006	10.1099/ijs.0.63802-0
	0.51%	Increase			Blast	Proteobacteria	Gamma-	Xanthomonadales	Rhodanobacteraceae	Dokdonella	sp.	HM488726.1	Aerobic	Yoon et al., 2006	10.1099/ijs.0.63802-0
					Blast	Proteobacteria	Gamma-	Xanthomonadales	Rhodanobacteraceae	Dokdonella	sp.	**LC025169.1**	Aerobic	Yoon et al., 2006	10.1099/ijs.0.63802-0
						TGGGGAATATTGGACAATGGGCGCAAGCCTGATCCAGCCATGCCGCGTGGGTGAAGAAGGCCTTCGGGTTGTAAAGCCCTTTTGTTCGGGAAGAAATCGTACGGGTGAATATCCCGTGCGGATGACGGTACCGGAAGAATAAGCACCGGCTAACTTCGTG									
**OTU_116**	**Relative abundance**	**Active layer**	**Intermediate layer**	**Permafrost layer**	**Assignment**	**Phylum**	**Class**	**Order**	**Family**	**Genus**	**Species**	**GenBank ID**	**Growth**	**Reference**	**DOI**
					RDP	Caldiserica	Caldisericia	Caldisericales	Caldisericaceae	Caldisericum	sp.		Anaerobic	Mori et al., 2009	10.1099/ijs.0.010033-0
	3.14%		Decrease		Blast	Caldiserica	Caldisericia	Caldisericales	Caldisericaceae	Caldisericum	sp.	KY926861.1	Anaerobic	Mori et al., 2009	10.1099/ijs.0.010033-0
						TGGGGAATCATGGTCAATGGGCGAAAGCCTGAACCTGCGACGCCGCGTGAGTGATGAAGGTCGTAAGATCGTAAAACTCTTTTCAGGGAGCTTAAAGCTCCGGCTCTAACAGAGCCTGGAGTATGACTATCCCTGGAATAAGCCCCAGCTAACTACGTG									
**OTU_6022**	**Relative abundance**	**Active layer**	**Intermediate layer**	**Permafrost layer**	**Assignment**	**Phylum**	**Class**	**Order**	**Family**	**Genus**	**Species**	**GenBank ID**	**Growth**	**Reference**	**DOI**
					RDP	Unclassified									
	0.73%			Increase	Blast	Aminicenantes	(bad alignment)					KU555233.1			
						TGGGGAATATTGCGCAATGGGCGAAAGCCTGACGCAGCAACGCCGCGTGGATGATGAAGGCCTTCGGGTCGTAAAATCCTGTTGTAGGGGACGAAACTTCGCGCAAGCGAACTGACGGTACCCTACGAGGAAGCCCCGGCTAACTACGTG									

Supplementary Table S3: **Differences of least-square means for soil variables**, across depths and treatments when justified by ANOVA results in Table 1; bold text denotes significant p-values ($p<0.05$). AL = Active layer; IL = Intermediate layer, PL = Permafrost layer, C = Control, DT = Deep-thaw

Organic matter content (OMC, square-root transformed)

**Depth**	estimate	SE	df	t.ratio	p.value
AL-IL	0.028	0.072	20	0.390	0.920
AL-PL	0.452	0.072	20	6.306	**<.0001**
PL-IL	-0.424	0.072	20	-5.916	**<.0001**

pH

**Depth**	estimate	SE	df	t.ratio	p.value
AL-IL	-0.169	0.134	20	-1.262	0.432
AL-PL	-0.415	0.134	20	-3.096	**0.015**
PL-IL	0.246	0.134	20	1.834	0.184

**Depth:Treatment**	estimate	SE	df	t.ratio	p.value
C_AL -C_IL	-0.358	0.190	20	-1.890	0.436
C_AL -C_PL	-0.905	0.190	20	-4.774	**0.001**
C_PL -C_IL	0.547	0.190	20	2.884	0.084
DT_AL -DT_IL	0.020	0.190	20	0.106	1.000
DT_AL -DT_PL	0.075	0.190	20	0.396	0.999
DT_PL -DT_IL	-0.055	0.190	20	-0.290	1.000
C_AL -DT_AL	-0.588	0.193	10	-3.041	0.096
C_IL -DT_IL	-0.210	0.193	10	-1.086	0.877
C_PL -DT_PL	0.392	0.193	10	2.025	0.392

Gravimetric water content

**Depth**	estimate	SE	df	t.ratio	p.value
AL-IL	-0.017	0.068	20	-0.246	0.967
AL-PL	0.223	0.068	20	3.290	**0.010**
PL-IL	-0.239	0.068	20	-3.536	**0.006**

Carbon content % (n = 3)

**ANOVA**	F.value	p.value
Treatment	1.153	0.343
Depth	11.837	**0.004**
Treatment:Depth	0.523	0.612

**Depth**	estimate	SE	df	t.ratio	p.value
AL-IL	-0.005	0.071	8	-0.064	0.998
AL-PL	0.299	0.071	8	4.181	**0.008**
PL-IL	0.303	0.071	8	4.245	**0.007**

Supplementary Table S4: Pairwise comparisons on the effects of treatment and depth on bacterial communities (manyglm). Statistics were obtained with 1000 sampling iterations using PIT-trap resampling, uncorrected P-values were obtained by re-leveling the interaction factor (5 times) and p-values were then corrected using Holm correction factor for the 25 pairwise comparisons (conservative correction: 15 non-redundant comparisons).

Baseline	Comparison	Wald value	Pr(>Wald)	Pr(>Wald) (holm-correction, n=25)
C_AL vs	Intercept	42.27	0.001	
	C_IL	19.43	0.001	**0.025**
	C_PL	20.77	0.001	**0.025**
	DT_AL	16.35	0.086	0.320
	DT_IL	16.32	0.057	0.306
	DT_PL	20.01	0.001	**0.025**
DT_AL vs	Intercept	37.42	0.001	
	C_AL	16.35	0.080	0.320
	C_IL	19.04	0.003	**0.039**
	C_PL	19.37	0.002	**0.030**
	DT_IL	11.57	0.821	1.000
	DT_PL	21.09	0.001	**0.025**
C_IL vs	Intercept	33.58	0.001	
	C_AL	19.43	0.002	**0.030**
	C_PL	14.62	0.018	0.126
	DT_AL	19.04	0.003	**0.039**
	DT_IL	17.86	0.004	**0.044**
	DT_PL	17.69	0.001	**0.025**
DT_IL vs	Intercept	33.35	0.001	
	C_AL	16.32	0.051	0.306
	C_IL	17.86	0.004	**0.044**
	C_PL	18.89	0.001	**0.025**
	DT_AL	11.57	0.837	1.000
	DT_PL	21.25	0.001	**0.025**
C_PL vs	Intercept	28.7	0.001	
	C_AL	20.77	0.001	**0.025**
	C_IL	14.62	0.013	0.104
	DT_AL	19.37	0.001	**0.025**
	DT_IL	18.89	0.001	**0.025**
	DT_PL	13.32	0.005	**0.045**

	Summary
Control Active Layer (C_AL)	**a**
Deep-Thaw Active Layer (DT_AL)	**a**
Control Intermediate Layer (C_IL)	b
Deep-Thaw Intermediate Layer (DT_IL)	**a**
Control Permafrost Layer (C_PL)	b
Deep-Thaw Permafrost Layer (DT_PL)	c

Supplementary Table S5: **Responses of bulk and intrinsic potential respiration to soil depth, treatment and temperature, and differences of least-square means** of respiration at 11ºC and 21ºC and its temperature sensitivity (Q_{10}); bold text denotes significant p-values ($p<0.05$). AL = Active layer; IL = Intermediate layer, PL = Permafrost layer, C = Control, DT = Deep-thaw, Temp = Temperature

	Decadal winter warming (treatment)		Depth		Treatment: Depth		Temperature		Depth: Temp		Treatment: Temp		Depth: Treatment: Temp	
Respiration rate	F	p	F	p	F	p	F	p	F	p	F	p	F	p
per g soil DW	5.852	**0.036**	49.307	**<0.001**	1.702	0.193	5.172	**0.028**	0.192	0.826	0.082	0.776	0.142	0.868
per g soil OM	0.259	0.622	1.040	0.361	8.977	**0.001**	18.907	**<0.001**	0.702	0.500	0.299	0.587	0.518	0.599

Bulk potential respiration (per g soil DW)

**Depth**	estimate	SE	df	t.ratio	p.value
AL-IL	0.178	0.241	48	0.740	0.741
AL-PL	2.110	0.233	48	9.049	**<.0001**
IL-PL	1.932	0.241	48	8.021	**<.0001**
**Treatment**	estimate	SE	df	t.ratio	p.value
C-DT	0.911	0.385	10	2.363	**0.040**

Intrinsic potential respiration (per g organic matter DW)

**Depth:Treatment**	estimate	SE	df	t.ratio	p.value
C_AL - C_IL	0.352	0.172	48.000	2.042	0.335
C_AL - C_PL	0.589	0.172	48.000	3.416	**0.016**
C_IL - C_PL	0.237	0.172	48.000	1.374	0.742
DT_AL - DT_IL	0.027	0.183	48.000	0.147	1.000
DT_AL - DT_PL	-0.425	0.172	48.000	-2.462	0.156
DT_IL - DT_PL	-0.451	0.183	48.000	-2.469	0.154
C_AL - DT_AL	0.530	0.208	10.000	2.543	0.199
C_IL - DT_IL	0.204	0.217	10.000	0.942	0.926
C_PL - DT_PL	-0.484	0.208	10.000	-2.323	0.269

Q10

**Depth**	estimate	SE	df	t.ratio	p.value
AL-IL	-0.192	0.128	20	-1.500	0.312
AL-PL	0.242	0.128	20	1.890	0.168
PL-IL	-0.435	0.128	20	-3.390	**0.008**
**Depth:Treatment**	estimate	SE	df	t.ratio	p.value
C_AL -C_IL	-0.039	0.181	20	-0.216	1.000
C_AL -C_PL	0.030	0.181	20	0.165	1.000
C_PL -C_IL	-0.069	0.181	20	-0.381	0.999
DT_AL -DT_IL	-0.345	0.181	20	-1.905	0.428
DT_AL -DT_PL	0.455	0.181	20	2.508	0.169
DT_PL -DT_IL	-0.800	0.181	20	-4.413	**0.003**
C_AL -DT_AL	-0.231	0.181	10	-1.276	0.791
C_IL -DT_IL	-0.538	0.181	10	-2.965	0.108
C_PL -DT_PL	0.193	0.181	10	1.067	0.884

Supplementary Table S6: Multiple regressions between (A) bulk and (B) intrinsic potential soil respiration at 11ºC and soil chemistry, bacterial community structure and root density, without (1), with (2) treatment and with its interactions (3) with the other independent predictors. Positive soil PC1 indicates lower pH, higher NH4+, organic matter content and soil moisture; all variables are scaled and centered before analyses. Models with bacterial Pco1 not including soil PC1 are also shown; bold values indicate slopes or intercepts significantly different from 0 (p<0.05); highlighted cells indicate the best fitting model(s) for bulk and intrinsic potential respiration

A.1

Predictors of bulk respiration (per g DW)	AICc	R^2	Variable	Effect size	
				Slope (±SE)	p
Soil	47.47	0.825	Soil PC1	0.893 ± 0.073	**<0.001**
Soil + Bacteria	40.46	0.879	Soil PC1	0.770 ± 0.073	**<0.001**
			Bacterial PCo1	-1.158 ± 0.347	**0.003**
Soil + Root density	48.47	0.834	Soil PC1	0.896 ± 0.072	**<0.001**
			Root density	0.094 ± 0.073	0.214
Root density + Soil + Bacteria	43.39	0.879	Soil PC1	0.770 ± 0.077	**<0.001**
			Root density	0.001 ± 0.075	0.985
			Bacterial PCo1	-1.155 ± 0.402	**0.009**
Bacteria	89.57	0.539	Bacterial PCo1	-3.032 ± 0.598	**<0.001**

B.1

Predictors of intrinsic respiration (per g SOM)	AICc	R^2	Variable	Effect size	
				Slope (±SE)	p
Soil	106.29	0.039	Soil PC1	0.192 ± 0.168	0.267
Soil + Bacteria	106.91	0.040	Soil PC1	0.172 ± 0.194	0.386
			Bacterial PCo1	-0.202 ± 0.938	0.832
Soil + Root density	108.61	0.051	Soil PC1	0.188 ± 0.170	0.280
			Root density	-0.107 ± 0.172	0.541
Root density + Soil + Bacteria	111.16	0.061	Soil PC1	0.125 ± 0.203	0.544
			Root density	-0.162 ± 0.199	0.423
			Bacterial PCo1	-0.624 ± 1.075	0.568
Bacteria	107.09	0.016	Bacterial PCo1	-0.597 ± 0.822	0.476

A.2

Predictors of bulk respiration (per g DW)	AICc	R^2	Variable	Effect size		Main deep-thaw effect	
				Slope (±SE)	p	Slope (±SE)	p
Soil + treatment	45.68	0.846	Soil PC1	0851 ± 0.072	**<0.001**	-0.302 ± 0.144	0.062
Soil + Bacteria + treatment	33.98	0.899	Soil PC1	0.715 ± 0.068	**<0.001**	-0.379 ± 0.120	**0.010**
			Bacterial PCo1	-1.271 ± 0.317	**0.001**		
Soil + Root density + treatment	43.81	0.866	Soil PC1	0.843 ± 0.068	**<0.001**	-0.391 ± 0.143	**0.021**
			Root density	0.149 ± 0.070	**0.045**		
Root density + Soil + Bacteria + treatment	36.59	0.901	Soil PC1	0.725 ± 0.070	**<0.001**	-0.400 ± 0.125	**0.010**
			Root density	0.047 ± 0.069	0.501		
			Bacterial PCo1	-1.158 ± 0.360	**0.004**		
Bacteria + treatment	83.97	0.537	Bacterial PCo1	-2.914 ± 0.577	**<0.001**	-0.778 ± 0.239	**0.009**

B.2

Predictors of intrinsic respiration (per g SOM)	AICc	R^2	Variable	Effect size		Main deep-thaw effect	
				Slope (±SE)	p	Slope (±SE)	p
Soil + treatment	108.88	0.043	Soil PC1	0.174 ± 0.178	0.339	-0.131 ± 0.355	0.721
Soil + Bacteria + treatment	111.73	0.045	Soil PC1	0.146 ± 0.207	0.490	-0.146 ± 0.365	0.697
			Bacterial PCo1	-0.263 ± 0.962	0.788		
Soil + Root density + treatment	111.50	0.052	Soil PC1	0.179 ± 0.180	0.332	-0.073 ± 0.376	0.851
			Root density	-0.097 ± 0.183	0.603		
Root density + Soil + Bacteria + treatment	114.26	0.063	Soil PC1	0.114 ± 0.213	0.597	-0.077 ± 0.380	0.843
			Root density	-0.152 ± 0.208	0.475		
			Bacterial PCo1	-0.628 ± 1.092	0.571		
Bacteria + treatment	109.35	0.030	Bacterial PCo1	-0.597 ± 0.829	0.479	-0.228 ± 0.344	0.523

A.3

Predictors of bulk respiration (per g DW)	AICc	R^2	Variable	Control samples		Deep-thaw samples		Deep-thaw (treatment) effect	
				Slope (±SE)	p	Slope (±SE)	p	Intercept (±SE)	p
Soil * treatment	48.30	0.848	Soil PC1	0.823 ± 0.074	**<0.001**	-0.892 ± 0.126	**<0.001**	-0.300 ± 0.146	0.066
(Soil + Bacteria) * Treatment	39.81	0.901	Soil PC1	0.687 ± 0.075	**<0.001**	0.755 ± 0.119	**<0.001**	-0.376 ± 0.123	**0.012**
			Bacterial PCo1	-1.176 ± 0.381	**0.011**	-1.344 ± 0.514	**0.028**		
(Soil + Root density) * Treatment	49.15	0.871	Soil PC1	0.776 ± 0.080	**<0.001**	0.896 ± 0.120	**<0.001**	-0.451 ± 0.168	**0.023**
			Root density	0.341 ± 0.235	0.177	0.140 ± 0.086	0.138		
(Root density + Soil + Bacteria) * Treatment	46.84	0.903	Soil PC1	0.687 ± 0.078	**<0.001**	0.775 ± 0.127	**<0.001**	-0.392 ± 0.156	**0.031**
			Root density	0.011 ± 0.243	0.964	0.052 ± 0.092	0.589		
			Bacterial PCo1	-1.165 ± 0.472	**0.036**	-1.169 ± 0.611	0.092		
Bacteria * treatment	86.82	0.538	Bacterial PCo1	-3.079 ± 0.772	**0.002**	-2.830 ± 0.811	**0.006**	-0.778 ± 0.243	**0.009**

B.3

Predictors of intrinsic respiration (per g SOM)	AICc	R^2	Variable	Control samples		Deep-thaw samples		Deep-thaw (treatment) effect	
				Slope (±SE)	p	Slope (±SE)	p	Intercept (±SE)	p
Soil * treatment	97.65	0.367	Soil PC1	0.716 ± 0.226	**0.009**	-0.445 ± 0.184	**0.036**	-0.161 ± 0.295	0.597
(Soil + Bacteria) * Treatment	93.33	0.539	Soil PC1	0.402 ± 0.240	0.125	-0.268 ± 0.182	0.176	-0.195 ± 0.265	0.477
			Bacterial PCo1	-2.826 ± 1.198	**0.040**	1.744 ± 0.787	0.054		
(Soil + Root density) * Treatment	101.93	0.408	Soil PC1	0.657 ± 0.247	**0.024**	-0.450 ± 0.178	**0.032**	-0.260 ± 0.358	0.484
			Root density	0.485 ± 0.723	0.518	-0.181 ± 0.128	0.192		
(Root density + Soil + Bacteria) * Treatment	100.16	0.549	Soil PC1	0.405 ± 0.245	0.133	-0.292 ± 0.195	0.172	0.054 ± 0.333	0.875
			Root density	-0.466 ± 0.766	0.558	-0.065 ± 0.141	0.656		
			Bacterial PCo1	-3.314 ± 1.463	**0.050**	1.524 ± 0.939	0.144		
Bacteria * treatment	92.45	0.456	Bacterial PCo1	-3.941 ± 1.051	**0.003**	2.251 ± 0.734	**0.012**	-0.228 ± 0.263	0.407
